# Betulin Complex in γ-Cyclodextrin Derivatives: Properties and Antineoplasic Activities in *In Vitro* and *In Vivo* Tumor Models

**DOI:** 10.3390/ijms131114992

**Published:** 2012-11-15

**Authors:** Codruta Şoica, Cristina Dehelean, Corina Danciu, Hai Ming Wang, Gerhard Wenz, Rita Ambrus, Florina Bojin, Mariana Anghel

**Affiliations:** 1 Faculty of Pharmacy, University of Medicine and Pharmacy, 2 Eftimie Murgu, Timisoara 300041, Romania; E-Mails: codrutasoica@umft.ro (C.S.); corina_tiulea@yahoo.com (C.D.); 2 Organic Macromolecular Chemistry, Saarland University, Campus Saarbrücken, Saarbrücken D-66123, Germany; E-Mails: lwplay@msn.com (H.M.W.); g.wenz@mx.uni-saarland.de (G.W.); 3 Institute of Pharmaceutical Technology, University of Szeged, 6 Eotvos ut., Szeged H-6720, Hungary; E-Mail: rita-techno@freemail.hu; 4 Faculty of Medicine, University of Medicine and Pharmacy, 2 Eftimie Murgu, Timisoara 300041, Romania; E-Mails: florinabojin@umft.ro (F.B.); biochimie@umft.ro (M.A.)

**Keywords:** melanoma, betulin, cyclodextrin, MTT, immunocytochemistry, C57BL/6J mice, antitumor activity

## Abstract

Given the present high incidence of melanoma and skin cancer, interest in potential drugs of plant origin has increased significantly. Pentacyclic lupane-type triterpenes are widely distributed in plants, offering numerous pharmacological benefits. Betulin is an important compound in the bark of *Betula pendula* Roth and has important therapeutic properties, including antitumor activities. Its biological effect is limited by its poor water solubility, which can be improved by cyclodextrin complexation. The best results have been obtained by using a novel cyclodextrin derivative, octakis-[6-deoxy-6-(2-sulfanyl ethanesulfonate)]-γ-CD. The complexes between betulin and the previously mentioned cyclodextrin were analyzed by scanning electron microscopy (SEM)and differential scanning calorimetry (DSC) and pharmacologically evaluated *in vitro* (MTT and immunocytochemistry tests) and *in vivo* in C57BL/6J mice. The solubility of betulin is improved by cyclodextrin complexation, which creates a stable complex that improves the *in vitro* and *in vivo* properties of the active compound.

## 1. Introduction

The interest in the development of drugs of plant origin to treat incurable diseases, such as cancer and AIDS, has increased in the last few years [1,2]. Due to the high incidence of melanoma and skin cancer, studies of new therapeutic agents are highly important.

Pentacyclic lupane-type triterpenes are widely distributed in various species of plants (*i.e.*, *Betula* sp.), and the most active are betulin (Bet) and betulinic acid (BA). In addition to the use as folk remedies by Native Americans [2], BA has been found to act against HIV [3] and melanoma [4]. BA can be easily obtained via oxidation from another major component of birch bark, Bet [5]; both substances have been intensively studied, revealing a great number of pharmacological benefits.

Bet, lup-20(29)-ene-3β,28-diol, is an abundant compound that can be easily isolated from the bark of *Betula pendula* Roth species. Bet plays an important role in the semi-synthesis of BA and other active compounds [5]. In addition, Bet has long been investigated for its therapeutic properties, specifically its anti-inflammatory and antitumor properties [6]. First, studies showed limited or no effects on cancer cells [7]. However, recent studies have demonstrated that Bet has an important cytotoxic effect on colorectal, breast, prostate and lung tumor cells [8] by inducing apoptosis [9]; therefore, it cannot be regarded as an inert precursor but as a valuable anticancer agent [9].

An important issue that limits Bet’s biological effects is poor water solubility [5]. A possible solution to this challenge is the use of hydrophilic carriers, such as cyclodextrins (CDs). CDs are oligosaccharides derived from starch, consisting of toroidal shapes and hydrophilic outer faces, that form inclusion complexes with a large number of hydrophobic active drugs, which are accommodated in the inner cavity of CDs. Recent studies [10] have shown that pentacyclic triterpenes form inclusion complexes with natural and semisynthetic CDs, and the best results are obtained by using γ-CD derivatives, such as hydroxypropyl-γ-CD. However, all of these complexes show very low stability constants (*K* = 47 M^−1^), which impedes the delivery of highly potent drugs to a particular destination in the body. One possibility to increase the binding ability of CDs is to extend the cavity by the attachment of hydrophobic substituents. Steffen *et al.*[11] synthesized a library of substituted per-6-thio-6-deoxy-CDs, which complex with even highly hydrophobic molecules, leading to highly water-soluble complexes.

The present work focuses on the preparation and characterization of inclusion complexes between Bet and a novel CD derivative, octakis-[6-deoxy-6-(2-sulfanyl ethanesulfonate)]-γ-CD (GCDG) [12], which has the best solubilizing properties for Bet [13]. The complexes were then tested both *in vitro* and *in vivo* using experimental animal models.

## 2. Results

### 2.1. SEM

**Figure 1:**
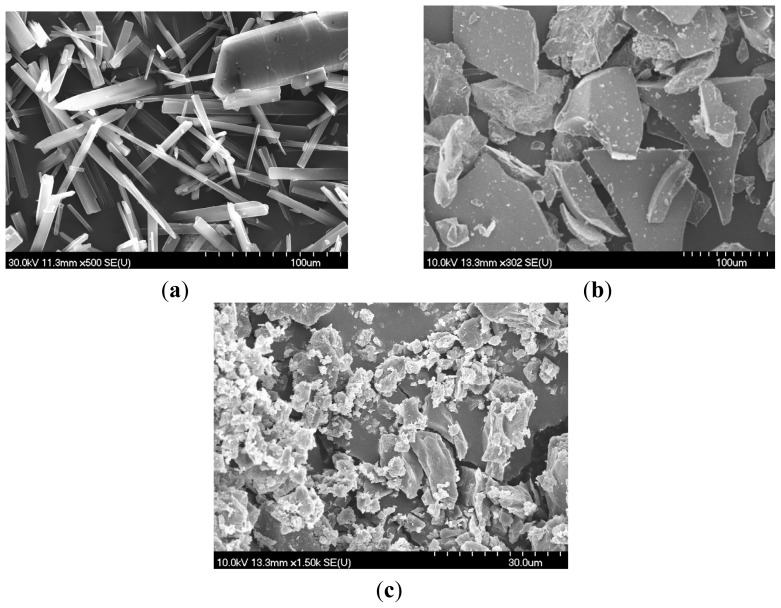
Scanning electron microscopy (SEM) pictures of: (**a**) betulin (Bet), (**b**) octakis-[6-deoxy-6-(2-sulfanyl ethanesulfonate)]-γ-CD (GCDG) and (**c**) Bet:GCDG 1:1 complex.

Scanning electron microscopy (SEM) was used to assess the microscopic aspects of the drug, the complexing agent and complex formation (Figure 1a–c).

Although this method does not completely confirm complex formation, it helps to identify the existence of a single component in the preparation products. In the SEM photographs, the pure drug, Bet (Figure 1a), is characterized by the presence of crystalline particles of a regular size and parallelepipedic shape. The physical mixture of Bet and GCDG showed a slight crystalline structure of both the drug and CD (not shown). Drug crystals mixed with complexing agent crystals adhered to the surfaces of each other. In contrast, a drastic change in the morphology and crystalline nature was observed in the 1:1 kneaded product (Figure 1c), revealing an apparent interaction in the solid state. The SEM photographs of the inclusion complexes prepared by the kneading method showed a characteristic morphology as the small-sized particles tended to aggregate, indicating the existence of an amorphous product with the presence of a single component in the complex, thus suggesting maximum complexation.

### 2.2. DSC

**Figure 2:**
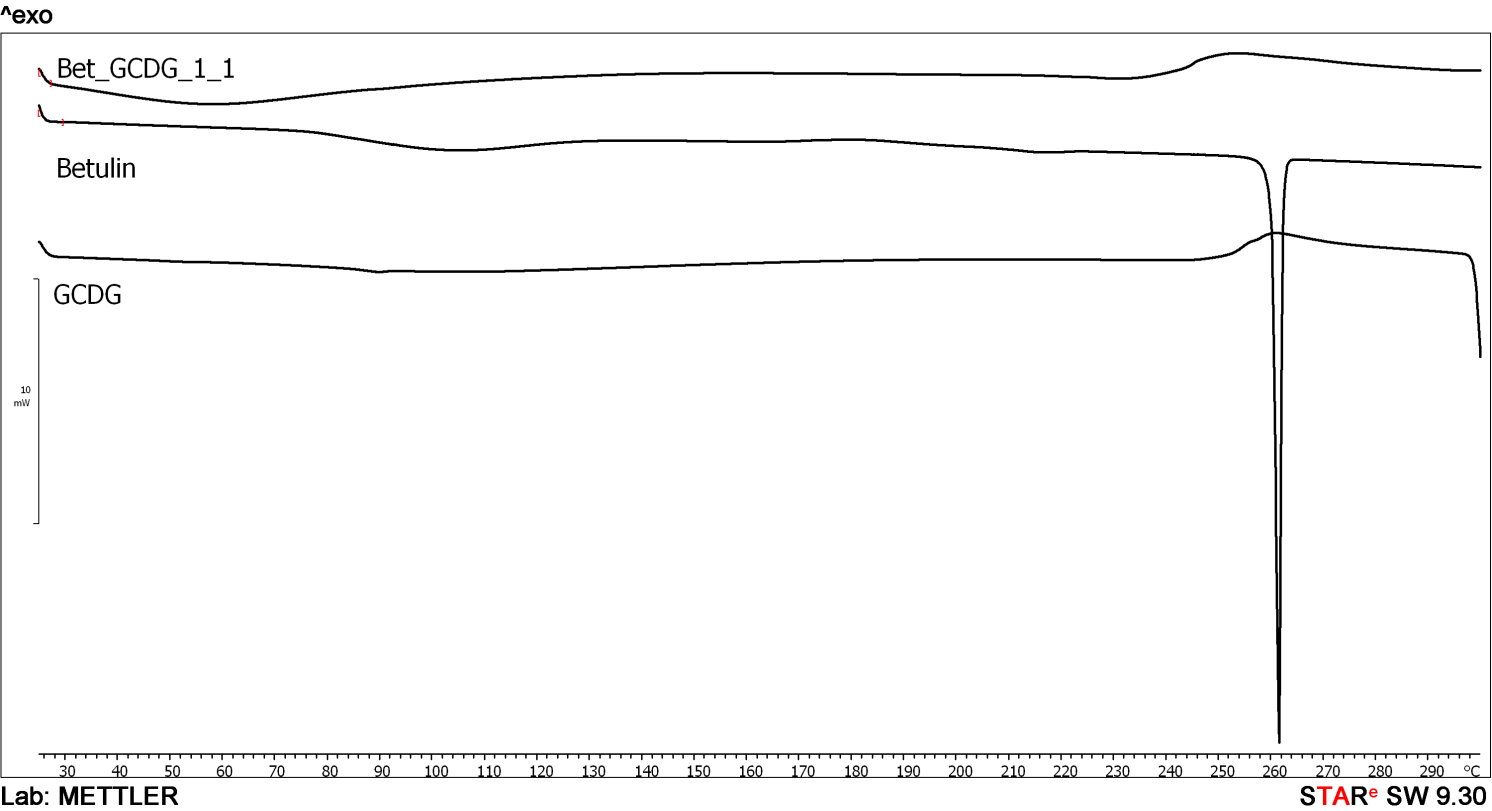
Differential scanning calorimetry (DSC) curves of Bet, GCDG and their 1:1 complex.

The differential scanning calorimetry (DSC) profile is depicted in Figure 2. For GCGD, the graph does not show any peaks within the temperature range of the study; however, its melting point started at 300 °C. Bet exhibits a single sharp endothermic peak at 260 °C, as determined by sublimation of the substance. The Bet-GCDG complex is similar to the CD curve, and the endothermic peak of Bet disappeared completely, indicating formation of a molecular complex.

### 2.3. MTT *in Vitro* Analysis

**Figure 3:**
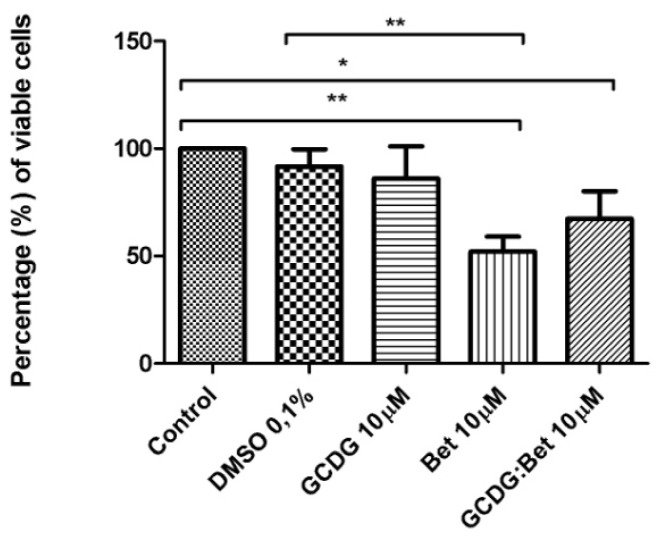
MTT assay showing the effects of GCDG, GCDG:Bet 1:1 and Bet on the viability of the B164A5 melanoma cell line. Viability is expressed as percentage of viable cells compared to the control (considered as 100%). DMSO was used to prepare stock solutions of the tested substances, and the highest DMSO concentration (0.1%) of the medium did not have any significant effect on cell proliferation. The dilution rate was 1:1000, the concentration of the stock solutions were 10 mM (Bet, GCDG, Bet:GCDG 1:1), and the Bet final concentration in the medium was 10 μM.

The complex activity was more intense compared with Bet alone in B164A5 cells, which can be observed in Figure 3.

Compared to the control, Bet and its complex with GCDG had important effects. Cyclodextrin alone had a small effect.

Note that the consecrated solvent used for testing active substances *in vitro* on different cell lines, DMSO, which is known for its neutral effects in a concentration less than or equal to 0.1%, had a very small effect on cellular viability (91% ± 0.11% viable cells). The results of the tested compounds from the assay were as follows: GCDG −88% ± 0.31% viable cells, Bet −52% ± 0.09% viable cells and Bet:GCDG 1:1 complex −62% ± 0.25% viable cells. The results are expressed as the mean ± standard deviation. One-way ANOVA followed by Bonferroni’s post-test was used to determine the significant differences between various experimental and control groups; *, ** and *** indicate *p* < 0.05, *p* < 0.01 and *p* < 0.001, respectively, compared with the control group.

### 2.4. Defining Mechanism-Based Toxicity

**Figure 4:**
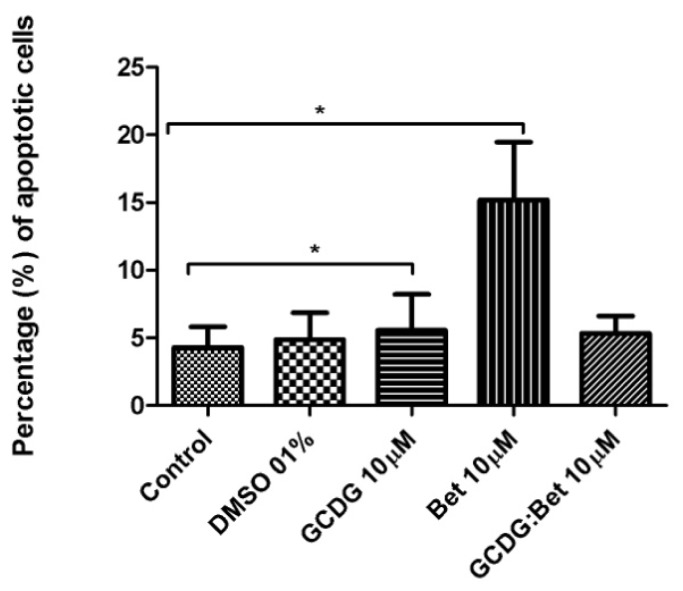
Percentage of apoptotic cells as shown by Annexin V/PI staining. The results are expressed as the mean ± standard deviation. One-way ANOVA followed by Bonferroni’s post-test was used to determine the significant difference between various experimental and control groups; *, ** and *** indicate *p* < 0.05, *p* < 0.01 and *p* < 0.001, respectively, compared with the control group.

**Figure 5:**
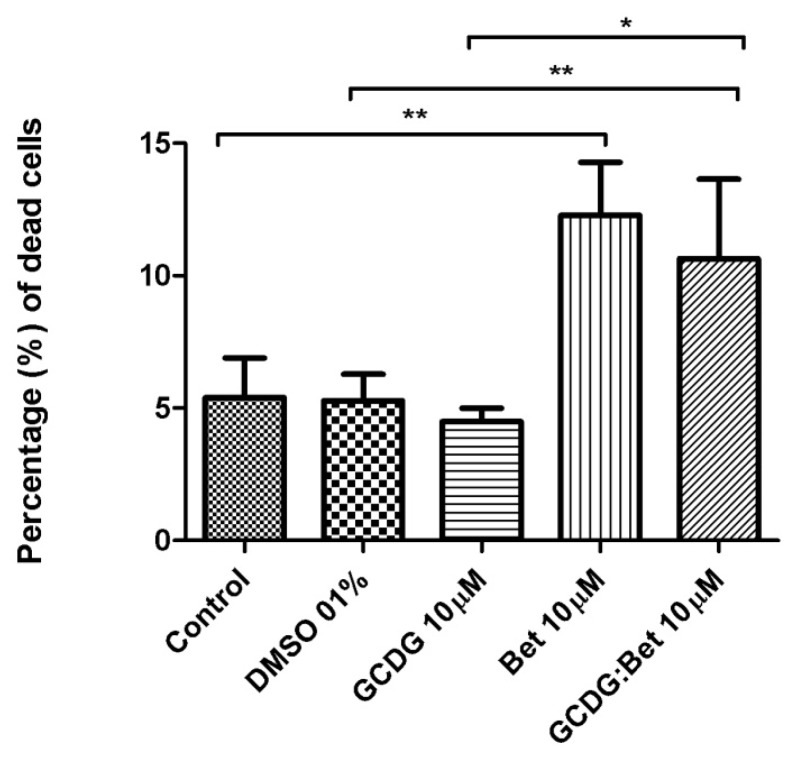
Percentage of dead cells as shown by Annexin V/PI staining. The results are expressed as the mean ± standard deviation. One-way ANOVA followed by Bonferroni’s post-test was used to determine the significant difference between various experimental and control groups; *, ** and *** indicate *p* < 0.05, *p* < 0.01 and *p* < 0.001, respectively, compared with the control group.

**Figure 6:**
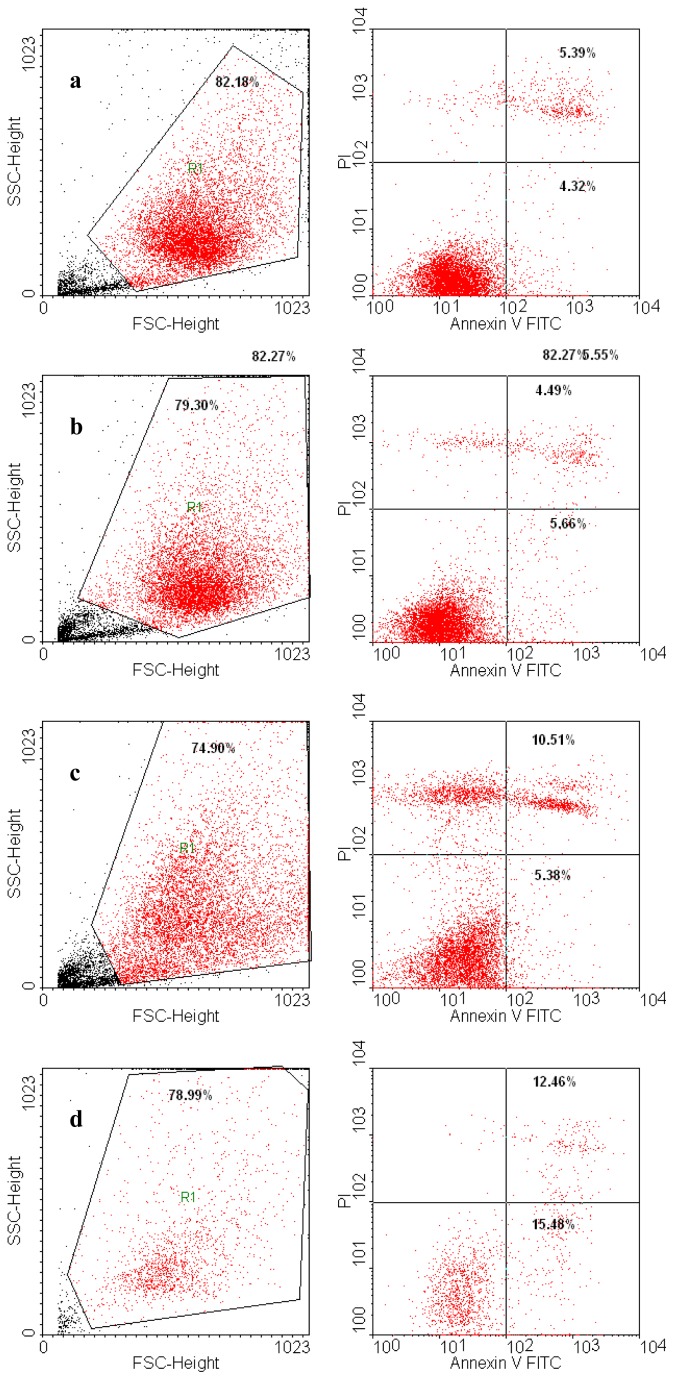
B164A5 melanoma cell line viability assay using Annexin V/PI. (**a**). Control; (**b**) GCDG; (**c**) Bet:GCDG 1:1 complex; (**d**) Bet.

**Figure 7:**
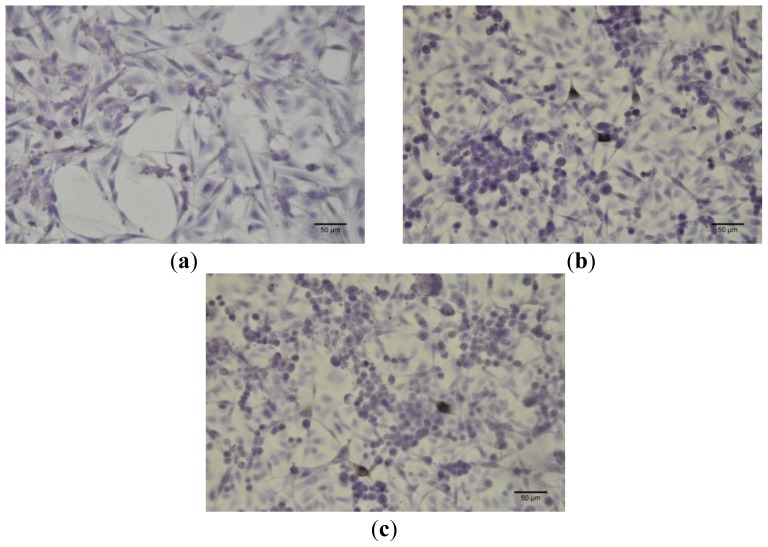
Expression of immunocytochemical markers in the B164A5 melanoma cell line. Although this cell line is a mixture of spindle-shaped and epithelial cells, they lacked expression of caspase 2 (**a**), cytokeratin 10 (**b**) and E-cadherin (**c**). Magnification 400×, scale bars 50 μm. Bet concentration, 10 μM.

**Figure 8:**
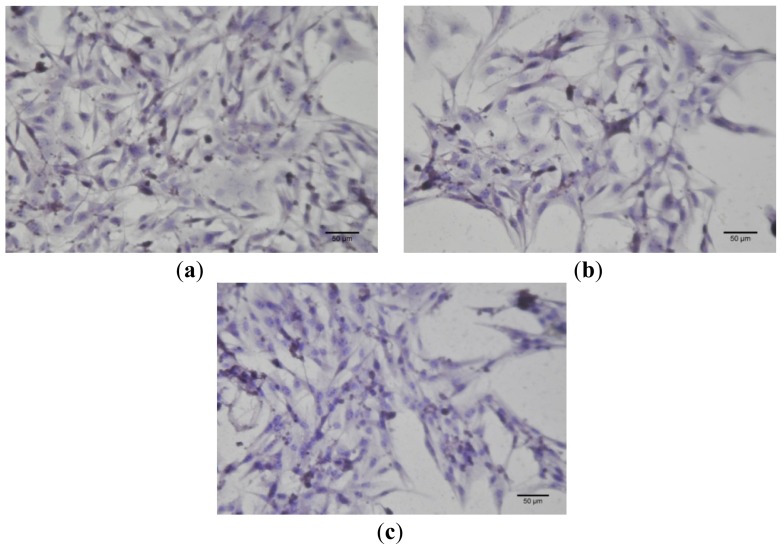
Caspase 2 expression in B16 4A5 cells treated with GCDG (**a**), GCDG:Bet 1:1 (**b**), and Bet alone (**c**). Bet concentration, 10 μM. Although the cellular number was markedly decreased when cells were treated with Bet, the caspase 2 pathway was not involved in this cellular death mechanism. Magnification 400×, scale bars 50 μm.

The results of the determination of the mechanism-based toxicity are presented in Table 1 and SI and Figures 4–8 and S1. The stock solution and dilutions were used as presented in the “Experimental section” and also applied in MTT tests (Bet concentration, 10 μM).

According to Figures 4 and 5, GCDG has almost no effect on the melanoma cell line B164A5, as the percentage of afflicted cells showed no significant difference compared with control cells.

The Bet:GCDG 1:1 complex-treated cells slightly increased in apoptosis, while the dead cells were almost double the control, revealing that complexation induced a decline of the initial Bet effect. Bet-treated cells have an increased percentage of affected cells, which were almost equally divided between apoptotic and dead cells.

Apoptotic cells (sub-G0 phase) were present in a larger proportion when the cells were treated with Bet alone or GCDG:Bet 1:1 complex.

The B164A5 melanoma cell line viability assay using Annexin V/PI showed different percentage values for dead and apoptotic cells in the viability assay using Annexin V/PI, with Bet inducing the highest amount of cellular death (Figure 6a–d).

Addition of Bet alone to the culture media at a ratio of 1:300 also induced a marked decrease in the total cell number. When complexed with GCDG, however, Bet had a very small effect on cellular viability, but a large proportion of the cells were only propidium iodide (PI) positive (Figure 6c), indicating that this complex destroyed cellular membranes and exposed the naked nucleus of the cells.

Histograms of cell cycle analysis for B164A5 cells treated with GCDG (a), GCDG:Bet 1:1 complex (b), and Bet alone (c) compared with the control (untreated cells), are depicted in Figure S1 (supporting information). Seventy-two h after cellular treatment, less cells were adherent to the culture flasks, but the regenerative potential was maintained.

Immunocytochemistry tests results are shown in Figures 7 and 8.

### 2.5. *In Vivo* Analysis

**Figure 9:**
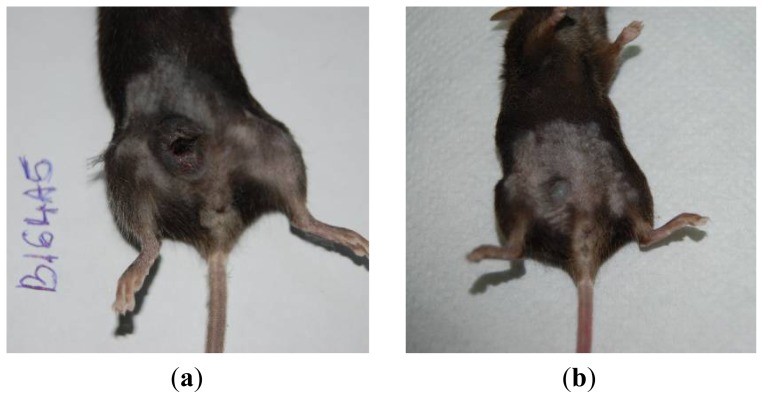
Macroscopic images of (**a**) untreated and (**b**) treated group of C57BL/6J mice at 16 days post-B164A5 tumor-cell inoculation. Note the reduction of the tumor in the treated group.

**Figure 10:**
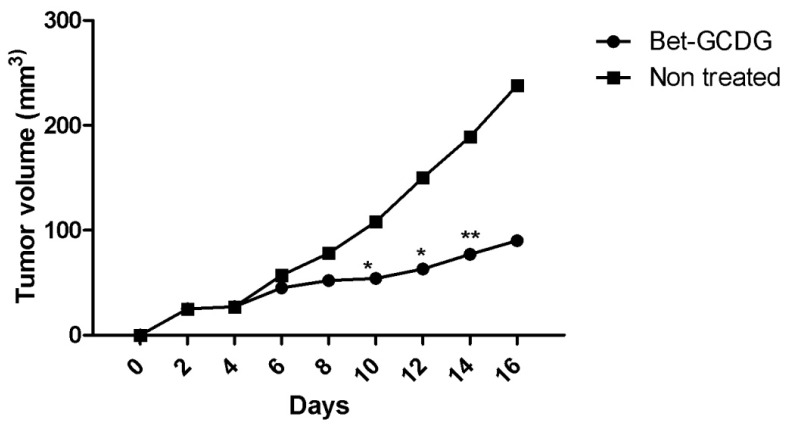
Tumor volume (mm^3^) for untreated or Bet:GCDG 1:1-treated mice, respectively. A paired Student’s *t*-test was used to compare the differences between untreated and treated mice; *, ** and *** indicate *p* < 0.05, *p* < 0.01 and *p* < 0.001, respectively, compared with the control group. For the subcutaneous injection with B16 cells in mice at a concentration of 0.5 × 10^5^ mL of cells without media, saline solution was used. At 2 days post-cell inoculation, the Bet-GCDG complex was administered intraperitoneally at a dose of 20 mg Bet per body weight daily for 14 days. After 16 days, the mice were sacrificed, and the pathological area was analyzed. Tumor size was measured every day using a caliper, and tumor volume was calculated as described by Eichenmuller *et al.*[14]. Body weights of the 2 groups of mice were recorded, and all mice were monitored during the whole experiment period.

**Figure 11:**
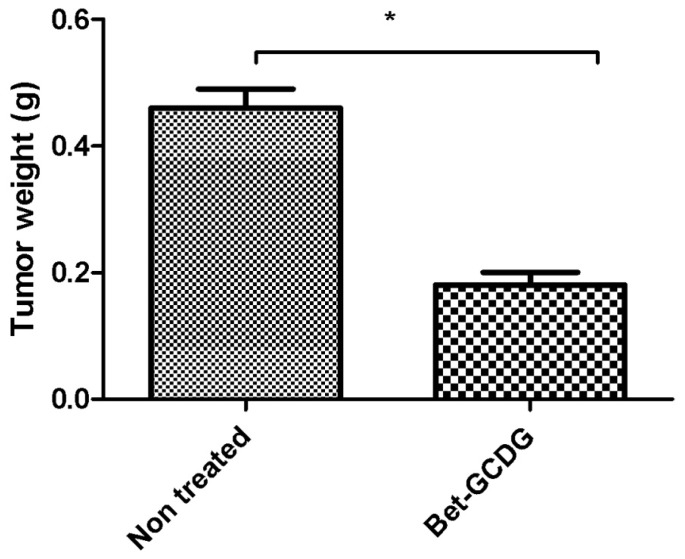
Tumor weight (g) for untreated and Bet:GCDG 1:1-treated mice. A paired Student’s *t*-test was used to compare the differences between untreated and treated mice; *, ** and *** indicate *p* < 0.05, *p* < 0.01 and *p* < 0.001, respectively, compared with the control group.

**Figure 12:**
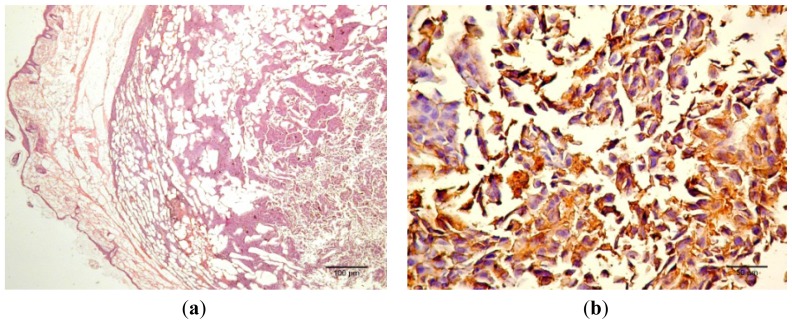

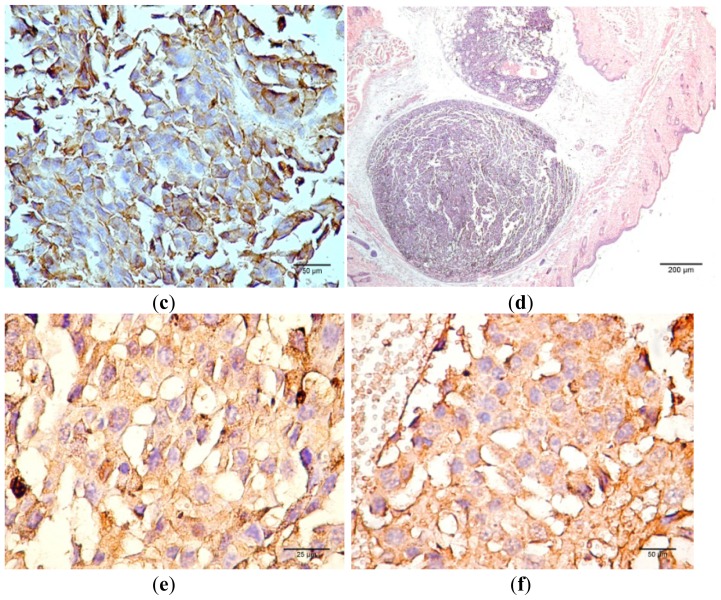
Morphology and immunohistochemistry of the studied melanoma samples. (**a**) HE of the untreatedgroup (scale bar 100 μm); (**b**) S100 for the untreated group (scale bar 50 μm); (**c**) VEGF for the untreated group (scale bar 50 μm); (**d**) HE of the treated group (scale bar 200 μm); (**e**) S100 for the treated group (scale bar 25 μm); (**f**) VEGF for the treated group (scale bar 50 μm).

Figures 9–12 and Table SII indicate the results of *in vivo* tests.

The tumor dimensions were visibly reduced after treatment with the Bet complex (Figures 9–11). The tumors were treated intraperitoneally with vehicle or with the Bet-GCDG complex in solution, and the tumor volume and weight were measured (Figures 10 and 11). The Bet-GCDG complex reduced the tumor volume significantly after 14 days of treatment, and the mean tumor weight was also drastically diminished.

Analyzing the *in vivo* data of the tumor dimensions during the 16 days indicated that GCDG-complexed Bet significantly reduced tumor growth.

VEGF and S100 expression (Figure 12) assessed in the *in vivo* data also indicated the positive activity of Bet *in vivo*. As mentioned in the *Experimental* section, uncomplexed Bet was not used for treatment because Bet alone is not soluble in water, and the aim of our research was to improve this deficiency.

The cord-like distribution of tumor cells (Figure 12a) differed from the compact appearance of the treated cells (Figure 12d). Note the predominance of pigmented cells in the second sample (Figure 12d, inset) compared with the first one (Figure 12a, inset). Staining for S100 protein was positive for both groups, but a more intense and homogenous reaction was registered for the first group (Figure 12b) compared with a moderate expression of S100 in the first line-group (Figure 12e). Additionally, differences in VEGF expression between the two groups were observed. Constant and homogenous VEGF expression characterized the first group with a cytoplasmic, granular pattern that was observed in most of the tumor cells (Figure 12c), while a low and inconstant expression characterized the second one, with a cytoplasmic, granular pattern in most of the tumor cells (Figure 12f).

## 3. Discussion

Bet is the most important compound in birch bark, which has been used in folk medicine for treatment of skin diseases [15], such as actinic keratoses and pre-carcinoma status of skin [16,17]. Important derivatives with an increased antitumor activity have been produced from Bet [15], which affects colorectal carcinoma (DLD-1), breast carcinoma (MCF7), prostate cancer (PC-3) and lung cancer (A549) [8]. In the case of A549 cells, apoptosis was induced [9]. Other studies revealed that Bet possesses low activity in several cell lines: melanoma (Mel-2), epidermoid carcinoma, leukemia and neuroblastoma [15]. The present study analyzed Bet antitumor activity in murine B164A5 melanoma cells and showed its therapeutic relevance, indicating that Bet can be effective in melanoma cells depending on their origin. Therefore, because Bet increased bioavailability, it could be used as a therapeutic agent in skin pathology.

The water solubility of pentacyclic triterpenes, such as Bet or BA, is very poor (only 0.02 μg/mL for BA) so they must be administered in animal models either via intraperitoneal or subcutaneous methods such as dispersed systems like nanoemulsions [18]. Changes in their formulation that enhance water solubility can increase bioavailability. Complexation with cyclodextrins is a well-known procedure to achieve this goal. Increasing the water solubility of these cholesterol-like triterpenoids by addition of hydrosoluble sections, such as sugar moieties at the C3 and/or C28 position of the lupane framework or synthesis of saponosides, led to potent pharmacological activity [19]. Natural and semisynthetic CDs have been involved in triterpene complexation [20] with good solubility but rather small stability constant values of the complexes, in which the best results were obtained with γ-CD derivatives. Higher binding constants in the range of 10^5^–10^7^ M^−1^ are necessary to deliver highly potent drugs to a particular destination in the body without dispersing along the way. One possibility to increase the binding ability of CDs is to extend the cavity by substituting the primary hydroxyls by thioether groups [12], with the resulting CD derivatives being capable of complexing with even very highly hydrophobic molecules [11]. These complexes are characterized by very high binding constants (>10^6^ M^−1^) and are potentially applicable for chemo- or photodynamic therapy in cancer [21]. Our previous studies [13] have shown a significant increase in water solubility of Bet and BA by inclusion complexation with octakis-[6-deoxy-6-(2-sulfanyl ethanesulfonate)]-γ-CD (GCDG), with the binding constants reaching the value of *K* = 6 × 10^7^ M^−1^.

Previous studies [22] have demonstrated the solubilization of birch bark extracts using beta-cyclodextrin derivatives without obtaining a real inclusion complex, possibly because the triterpene molecule was too large to fit in the beta-cyclodextrin cavity. The external association between triterpenes and cyclodextrins increases water solubility for the active substances, but the best results are achieved by preparing real inclusion complexes. Therefore, our study works with a gamma-cyclodextrin derivative, which has an inner cavity that in our opinion allows the entrance of the triterpene molecule.

DSC analysis offers direct information about the thermal stability/phase transitions of CD complexes [23]. In combination with other methods, DSC analysis provides experimental evidence for the formation of CD-drug complexes and their stoichiometry in solution or solid state. DSC was used by Naidu *et al.*[24] to confirm the formation of meloxicam-CD binary systems. Similar conclusions were drawn by Mura *et al.*[25] in the study of CD-naproxen products. Liu *et al.*[26] used DSC to analyze CD complexes of an anticancer drug paclitaxel. In our previous studies, we also used DSC to characterize triterpene-CD complexes [27].

The endothermal peak of Bet indicates a sublimation process characteristic for the compound, previously confirmed by other thermal techniques [28]. The disappearance of the sublimation peak in the presence of the GCDG indicates that the active substance has been trapped in the cavity of the CD, and the sublimation properties are completely blocked in the studied temperature range. This behavior is direct evidence of the formation of a real inclusion complex.

Bet exhibits an important activity in B164A5 cells. Bet binds to the melanocortin receptors and promotes cell death in mouse melanoma cells [29]. Bet is still being investigated as an antitumor agent.

The B164A5/C57BL/6J mouse melanoma model has been used to evaluate chemotherapeutic agent activity because of the effects on the skin. B164A5 cells can be cultured *in vitro* and then transferred to the mouse body, specifically in C57BL/6J mice, which shows a high degree of acceptance. The inhibition of proliferation of B16 cells in the mouse, including tumor dimensions and histological evaluations, can be an indicator of treatment efficacy. Previous studies used B16 cells and C57BL/6J mice to test active compounds, such as dietary glycine [30]. A procedure was used in which the cells were first cultured and prepared *in vitro* in a proper medium, and then they were inoculated and transferred subcutaneously into the mouse [30]. The cell concentration is generally 10^5^ or 10^6^ mL^−1^, which leads to a model of tumor metastasis in a few weeks. A proper and reproducible model is helpful in testing pathological progression and the activity of chemotherapeutic agents. The aim of the present study was to obtain an experimental melanoma model and to evaluate the pathology after the application of a new formulation with Bet.

Figure 12 presents the progression of melanoma *in vivo* after the application of treatment (Bet and GCDG) at the end of experimental day 16. The HE images indicate a reduction in melanoma progression in the treated group. In the untreated group (group 1), two patterns of cutaneous melanoma were detected compared to the treated group (group 2) where there is only one pattern. In group 1, multiple necrosis, an oval tumor with a clear delineation and compact carcinoma beaches with vascular structures lacking endothelium and pigmented cells, was present. Group 2 was characterized by a reduced volume of cutaneous melanoma and no sign of affecting neighboring organs due to invasive cells. S100 and VEGF expression was more reduced in group 2 compared to group 1.

The caspase activity was upregulated neither by Bet nor by the complex suggesting a different mechanism of tumor cell death. Betulin derivative compounds upregulate caspases 8 and 9 in the HT-29 colon cancer cell line, which was not tested for Bet activity alone [31]. In addition, Bet-induced apoptosis does not involve the death receptor pathway but is dependent on mitochondria. Cytochrome c release and caspase activation occur independently of the Bcl-2 family proteins. [9]. Some previous studies [32,33] have shown that Bet apoptotic activity involves the sequential activation of caspase-9 and caspase-3/-7 and the cleavage of poly(ADP-ribose)polymerase (PARP). In another study [34], Bet pretreatment inhibited cadmium-induced apoptosis through an incomplete blockage incaspase-9 or -3-activation. Our study has clearly demonstrated that the Bet mechanism of action does not involve caspase-2 regulation.

C57BL/6J mice and others that have a common strain, B6D2F1, were used for a syngeneic tumor model with B16F10 cells [30,35]. The B16 melanoma model has been extensively used to analyze therapeutic agents for impact in this pathology [30].

Even though Bet is not as extensively used in melanoma treatment as BA [36], the present study claims its applicability as a therapeutic agent in this pathology if it is applied in a hydrophylic formulation. Application of the active compound at the beginning of the experiment for subcutaneous development of pathology determines an important reduction of pathological effects at the skin level. There are also some studies in which birch bark extract (with Bet as main component) was applied on mice with induced mutagenesis [37] or was analyzed pharmacokinetically [38]. Bet, like other pentacyclic triterpenes, is also important due to its lack of toxicity [38]. Because both the *in vitro* and *in vivo* activities of Bet are more effective as a GCDG complex, the mechanism of action likely occurs via direct action. Generally, small tumors (<2 mm diameter) are not dependent on angiogenesis. After experimental day 16, the tumors of the untreated group were larger than the ones of the other group. In addition, B16 tumor growth *in vivo* is dependent on vascularization. Tumor growth is slow and linear before vascularization but rapidly increases after blood vessel contribution occurs [30]. This tumor growth indicates a possible inhibition of neovascularization and the anti-angiogenic *in vivo* activity of Bet. Additionally, previous studies on embrionated eggs confirmed the anti-angiogenic character of Bet [39].

A possible mechanism of action of Bet may be its transformation into BA at the cellular level. This transformation may occur in the mitochondrial microenvironment, which contains several oxygen radicals that could induce Bet oxidation. Entrapment into cyclodextrins may influence this transformation. Future studies may quantify both plasmatic Bet and BA using electrospray liquid chromatography/mass spectrometry as previously applied for the BA alone [40]. VEGF and S100 expression were very intense for the untreated group compared with the treatment group, which supports the conclusion that Bet acts as an antitumor and anti-angiogenic agent as well as an antimelanoma agent. Tumor progression in terms of size and volume confirms the intervention of Bet on melanoma development. Proper formulations such as solutions are easily applied to the parenteral route. The preparation of solutions with an increased capacity to deliver an active agent in an emergency using fast routes, such as intraperitoneal methods, is an important goal of pharmaceutical technology. The high potency of Bet on the *in vivo* model illustrates its efficacy as a hydrosoluble agent in skin melanoma.

## 4. Experimental Section

Octakis-[6-deoxy-6-(2-sulfanyl ethanesulfonate)]-γ-CD (GCDG) was synthesized at the University of Saarland as previously published [11]. Betulin was purchased from Sigma Aldrich Ltd. (Taufkirchen, Germany, purity over 99%) and used as received. All other reagents were purchased and used as received.

### 4.1. Preparation of Complexes

Complexes were prepared using the kneading method: physical mixtures of Bet and GCDG were mixed with the same quantity of a solvent mixture consisting of ethanol and water (1:1). They were kneaded continuously until the bulk of the solvent mixture had evaporated; the mixture was then dried at room temperature (25 °C, normal atmospheric pressure) for 24 h, and then it was put in the oven at 105 °C for 7 h until constant weight was reached. The final product was pulverized and sieved through a 100-μm sieve. The binary products were prepared using a molar ratio of 1:1. The water solubility of the complex expressed as Bet concentration is 4.45 mM as previously determined whereas the cyclodextrin is highly soluble in water [13].

### 4.2. Scanning Electron Microscopy (SEM)

The crystal morphology was examined by scanning electron microscopy (Hitachi S4700, Hitachi Scientific Ltd., Japan). A sputter coating apparatus (Bio-Rad SC 502, VG Microtech, UK) was used to induce electric conductivity on the surface of the samples. The air pressure was 1.3–13.0 mPa. The samples were fixed onto a metallic stub with double-sided conductive tape (diameter 12 mm, Oxon, Oxford Instruments, UK). Images were taken in secondary electron image mode at 10 kV acceleration voltage.

### 4.3. Differential Scanning Calorimetry (DSC)

DSC was performed using a Mettler Toledo STAR Thermal Analysis System, DSC 821 (Switzerland). Argon was used as the carrier gas, the heating rate was 5 °C/min and the sample weight was 2–5 mg. Examinations were recorded from 25 °C up to 300 °C.

### 4.4. MTT *in Vitro* Analysis

The B164A5 cell line (ECACC; Sigma Aldrich origin Japan stored UK) was seeded onto a 96-well microplate and attached to the bottom of the well overnight. After 24 h, 200 μL of new medium containing Dulbecco’s Modified Eagle’s Medium (DMEM; Gibco BRL, Invitrogen, Carlsbad, CA, USA) and the tested substances were added and incubated for 72 h; the medium was supplemented with 10% fetal calf serum (FCS; PromoCell, Heidelberg, Germany) and 1% penicillin/streptomycin mixture (Pen/Strep, 10,000 IU/mL; PromoCell, Heidelberg, Germany). Melanoma cells were passaged at confluence after treatment with 5 mM EDTA; the living cells were then assayed by the addition of 20 μL of 5 mg/mL MTT solution. The intact mitochondrial reductase converted and precipitated MTT as blue crystals during a 4-h contact period. The medium was then removed, and the precipitated crystals were dissolved in 100 μL of dimethyl sulfoxide (DMSO; Sigma-Aldrich, Ayrshire, UK). Finally, the reduced MTT was spectrophotometrically analyzed at 545 nm using a microplate reader; wells with untreated cells were used as controls. All *in vitro* experiments were performed on two microplates with at least five parallel wells. DMSO was used to prepare stock solutions of the tested substances, and the highest DMSO concentration (0.1%) of the medium did not have any significant effect on cell proliferation. The dilution rate was 1:1000, and the concentration of stock solution was 10 mM.

### 4.5. Cell Culture Immunocytochemistry

Immunocytochemistry was performed for B164A5 cells (ECACC; Sigma Aldrich origin Japan stored UK) plated at a density of 10,000 cells/cm^2^ in 4-well glass chamber slides (Nalgene Nunc International, New York, NY, USA) and expanded for 24 h in culture medium. Bet, GCDG and Bet:GCDG 1:1 complexes were added to the culture media so that the dilution ratio was 1:300 and 1:1000, respectively. The control well contained untreated cells. Cells were maintained in culture for 72 h after addition of the substances and then prepared for immunocytochemical staining. After removing the culture medium, cells were washed and fixed with 4% paraformaldehyde (Sigma-Aldrich), permeabilized with 0.1% Triton X-100 (Sigma-Aldrich) and then analyzed for the expression of the proteins of interest, such as anti-h/m Caspase 2 (mCaspase 2 affinity purified rabbit IgG) (R & D Systems, Abingdon, UK). Untreated cells were also stained for the expression of cytokeratin 10 (mouse monoclonal IgG1, Santa Cruz Biotechnology, USA) and E-cadherin (mouse monoclonal IgG1, Santa Cruz Biotechnology). The staining protocol used the secondary biotinylated antibody binding and substrate addition (AEC) (Dako EnVision™ + System-HRP, Dako, CA, USA) following the manufacturer’s protocol. After counterstaining with hematoxylin solution (Hematoxylin, Mayer’s Lillie’s Modification, Dako) for 30 s and washing with tap water, the slides were mounted in an aqueous mounting media (Crystal/Mount™, Biomeda, CA, USA). Microscopy analysis was performed on a Nikon Eclipse E800 microscope.

### 4.6. Annexin V/PI Assay

Annexin V-FITC (Miltenyi Biotec, Gladbach, Germany) was used in cell death flowcytometric studies (apoptosis) combined with propidium iodide staining solution (BD Biosciences, San Jose, CA, USA) following the manufacturer’s protocol. Briefly, 10^6^ cells were washed in 1 × Annexin V Binding Buffer (BD Pharmigen), centrifuged at 300 g for 10 min, resuspended in the same solution and incubated with 10 μL of Annexin V-FITC for 15 min in the dark. After washing the cells with 1 mL specific binding buffer and centrifugation, the cell pellet was resuspended in 500 μL binding buffer, and 1 μg/mL of PI solution was added immediately prior to analysis by flow cytometry.

### 4.7. Cell Cycle Test

B164A5 cells (ECACC; Sigma Aldrich origin Japan stored UK) including control, betulin, GCDG-complexed betulin and GCDG-treated were submitted to cell cycle analysis using the CycleTest™ Plus, DNA Reagent Kit (Becton-Dickinson, San Jose, CA, USA). This method involves dissolving cell membrane lipids, eliminating cellular cytoskeleton and nuclear proteins, digestion of cell RNA, and stabilization of nuclear chromatin followed by propidium iodide (PI) binding to the clean, isolated nuclei. All procedures were performed according to the manufacturer’s protocols and included a first step of addition of 250 μL of Solution A (trypsin buffer), which was incubated for 10 min at room temperature. For the second step, 200 μL of Solution B (trypsin inhibitor and RNase buffer) was added and incubated for 10 min at room temperature. For the last step, 200 μL of cold (2–8 °C) Solution C (propidium iodide stain solution) was added to the same tube, gently mixed and incubated for 10 min in the dark in the refrigerator.

Data acquisition for the Annexin V/PI assay and cell cycle flow cytometric procedures was performed on a four-color capable FACSCalibur (Becton-Dickinson) flow cytometer, and the data were analyzed with the CellQuest Pro software (Becton-Dickinson).

### 4.8. Syngeneic Tumor Model

#### Ethics Statement

The experiment was analyzed and approved by the Bioethics Committee of the University of Medicine and Pharmacy “Victor Babes” Timisoara, Romania. The work protocol followed all of the NIAH-National Institute of Animal Health rules: animals were maintained during the experiment in standard conditions: 12 h light-dark cycle, food and water *ad libitum*, temperature 24 °C, and humidity above 55%. At the end of the experiment, animals were sacrificed by cervical dislocation.

Male C57BL/6J mice (eight weeks old) were purchased from Charles River (Sulzfeld, Germany). Five males were assigned to group 1 and were treated with GCDG and solvent (saline solution), and a second group of five males (group 2) was treated with the Bet-GCDG complex. B164A5 (ECACC and Sigma Aldrich, origin Japan stored UK) cells were grown as described in the MTT assay protocol. For the subcutaneous injection with B16 cells in mice at a concentration of 0.5 × 10^5^ mL of cells without media, saline solution was used. After two days post-cell inoculation, the Bet-GCDG complex was administered intraperitoneally at a dose of 20 mg Bet per body weight daily for 14 days. After 16 days the mice were sacrificed and the pathological area was analyzed. The dose of Bet was determined based on a corresponding dose of BA, a related compound. Tumor size was measured every day using a caliper, and tumor volume was calculated as described by Eichenmuller *et al.*[14]. Body weights of the two groups of mice were recorded throughout the experiment, and all mice were monitored during the whole experiment period. Because Bet is insoluble in water, it could not be used alone for such applications.

### 4.9. Histology, S100 and VEGF Expression

The skin samples analyzed by the haematoxilin-eosin (HE) technique demonstrate the presence of conjunctive-subepithelial skin carcinoma with the capability of dermis and adipose tissue invasion in the cancer group. The healthy skin shows a normal aspect with no major changes. For the treatment group, the only detectable changes were a decrease in hair follicle number, altered morphology of the skin layers and an increase in dermal collagen fiber density. For the cancer group, a VEGF and S100 expression analysis was performed, which indicated the pronounced intensity of VEGF and S100 expression on the sub-epithelial area of the skin and the decrease in VEGF expression distant from the epithelium.

### 4.10. Statistical Analysis

All experiments were performed in triplicate. The results were expressed as the mean ± standard deviation, and the differences among the means were evaluated using the one-way ANOVA test followed by Bonferroni’s post-test, with the level of statistical significance set at <0.05.

## 5. Conclusions

Bet is an active compound in murine melanoma. The solubility of Bet is improved by cyclodextrin complexation; this stable complex augments the *in vitro* and *in vivo* properties of the active compound. An increase in hydrosolubility demonstrates an important improvement for Bet antitumor activity. By complexation with suitable cyclodextrins, a parenteral formulation with a rapid effect can be prepared. The reduction in tumor size after Bet application was attributed to its anti-angiogenic effect.

## Supplementary Information



## Figures and Tables

**Table 1:** Evaluation of the variation between cell cycle phases between control cells and B164A5 melanoma cells treated with GCDG, Bet and their 1:1 complex.

Sample (10 μM)	Cell Cycle Phases			

	sub-G0 (%)	G0/G1 (%)	S (%)	G2/M (%)
Control	0.49 ± 0.02	79.65 ± 3.99	6.56 ± 0.16	10.01 ± 0.26
DMSO	0.73 ± 0.04	76.08 ± 2.21	11.54 ± 0.26	11.65 ± 0.18
GCDG	0.91 ± 0.04	79.42 ± 3.82	8.54 ± 0.18	8.54 ± 0.16
Bet:GCDG 1:1 complex	2.27 ± 0.05	82.76 ± 4.01	4.81 ± 0.15	8.41 ± 0.17
Bet	1.21 ± 0.03	67.83 ± 3.75	15.09 ± 0.31	10.39 ± 0.27
