# Supplementary material for: Betulin Complex in γ-Cyclodextrin Derivatives: Properties and Antineoplasic Activities in In Vitro and In Vivo Tumor Models
Source: Int J Mol Sci. 2012 Nov 15;13(11):14992–5011. doi: 10.3390/ijms131114992 (PMC3509624; doi:10.3390/ijms131114992)
Supplement: Supplementary file 1 [file ijms-13-14992-s001.pdf]

Supplymentary Information

Figure S1. Histograms of Cycle test analysis for B16 4A5 cells treated with GCDG (**a**), GCDG:Bet 1:1 complex (**b**) and Bet alone (**c**) compared with the control (untreated cells).

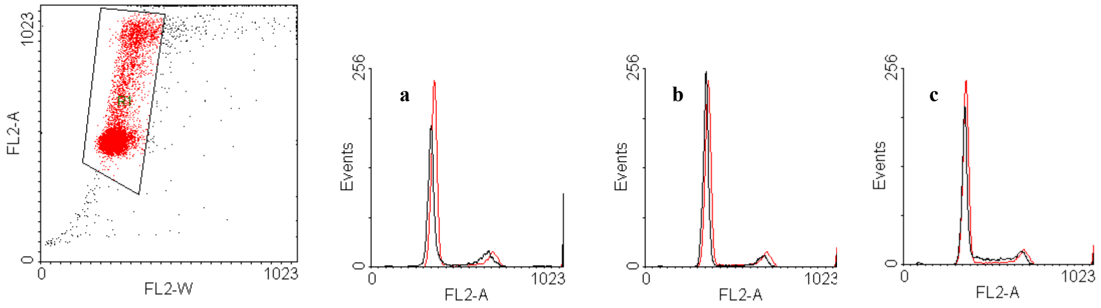

Table SI. Percentage of afflicted cells when treated with Bet in different combinations, compared with control.

Cellular Type	Substance (10 µM)	Afflicted Cells	
		**Apoptotic cells (%)**	**Dead cells (%)**
B16 4A5	Control	4.29 ± 0.18	5.39 ± 0.24
B16 4A5	DMSO	4.88 ± 0.19	5.28 ± 0.19
B16 4A5	GCDG	5.58 ± 0.21	4.49 ± 0.16
B16 4A5	Bet:GCDG 1:1 complex	5.36 ± 0.17	10.65 ± 0.29
B16 4A5	Bet	15.23 ± 0.34	12.28 ± 0.26

Table SII. Mean tumor weight for the 2 groups.

Mice Group	Wet Tumor Weight (g)
Non treated (vehicle)	0.46 ± 0.02
Treated with Bet complex	0.18 ± 0.01

© 2012 by the authors; licensee MDPI, Basel, Switzerland. This article is an open access article distributed under the terms and conditions of the Creative Commons Attribution license (http://creativecommons.org/licenses/by/3.0/).
